# Lymphoepithelial Cyst of the Pancreas: Serum Markers do not Help

**DOI:** 10.1155/1998/57832

**Published:** 1998-12

**Authors:** S. P. Chan, C. W. Hatton, G. L. Falk

**Affiliations:** 1 Department of Upper Gastro-intestinal Surgery Repatriation General Hospital Concord Sydney Australia; 2 Kiama New South Wales Australia; 3 PO Box 1085 Strathfield NSW 2135 Australia

## Abstract

We report a case of lymphoepithelial cyst of the pancreas with non-specific elevation of CA 19.9 and CEA. Pre-operative diagnosis by conventional means proved elusive, and only surgical resection and histopathology revealed the diagnosis. The origin and diagnosis are discussed by literature review.


*HPB Surgery*, 1998, Vol. 11, pp. 121-124
Reprints available directly from the publisher
Photocopying permitted by license only

© 1998 OPA (Overseas Publishers Association) N.V.
Published by license under
the Harwood Academic Publishers imprint,
part of The Gordon and Breach Publishing Group.
Printed in Malaysia.

*Case Report*

### Lymphoepithelial Cyst of the Pancreas: Serum Markers do not Help

S. P. CHAN, C. W. HATTON* and G. L. FALK†

*Department of Upper Gastro-intestinal Surgery, Repatriation General Hospital Concord, Sydney, Australia*

*(Received 20 October 1997)*

**We report a case of lymphoepithelial cyst of the pancreas with non-specific elevation of CA 19.9 and CEA. Pre-operative diagnosis by conventional means proved elusive, and only surgical resection and histopathology revealed the diagnosis. The origin and diagnosis are discussed by literature review.**

*Keywords:* Pancreas, cyst lymphoepithelial cyst, serum marker

### INTRODUCTION

Cystic lesions of the pancreas are uncommon and can be difficult to diagnose pre-operatively [6, 15]. The differential diagnosis of malignancy is ever present and requires assessment. We report a case of a lymphoepithelial cyst of the pancreas which was not correctly diagnosed on serum markers or radiology and laparotomy was necessary to obtain a conclusive diagnosis.

### CASE REPORT

The patient is a 47 year old male journalist. He presented with a 5 month history of back pain. He had previous multiple episodes of biliary pain but no pain to suggest an episode of acute pancreatitis. There was no history of fevers, jaundice of weight loss. He had a history of heavy alcohol intake and grew up on a sheep farm. Physical examination was normal.

Full Blood Count, electrolytes, Liver Function Tests and Serum Amylase were all normal. Serum tumour markers were also performed. Carbohydrate antigenic determinant (CA 19.9) was slightly elevated at 46 IU/mL (0-37 IU/mL). Carcinoembryonic antigen (CEA) was also elevated at 4.5 ng/mL (<3 ng/mL). CA125 was normal at 7 IU/mL (0-35 IU/mL). Hepatitis serology was negative.

* Gastroenterologist, Kiama, New South Wales.
† Address for correspondence: PO Box 1085, Strathfield NSW 2135, Australia.

An Abdominal Ultrasound showed a contracted gallbladder with multiple calculi, a hydatid cyst of the liver and a well-defined 46 mm homogenous mass in the head of the pancreas which was reported as a pseudocyst. Computerised tomography (CT) of the abdomen demonstrated a low density, well-defined lesion (measuring 51 mm × 35 mm) between the head of the pancreas and the vena cava. The rest of the pancreas imaged normally and there was no dilatation of the pancreatic duct or the billiary tree (Figs. 1 and 2). An endoscopic retrograde cholangiopancreatogram (ERCP) was performed to assess the pancreatic duct. The pancreatic duct was not cannulated successfully.

A CT-guided fine needle biopsy of the pancreatic cyst was undertaken. This also did not contribute towards a pre-operative diagnosis as no cellular material was obtained for assessment and insufficient cyst fluid was aspirated for tumour marker studies.

An upper midline laparotomy was performed. There was a 5 cm multilobulated soft cystic mass behind the bile duct and pancreatic head. The cyst was easily dissected from the pancreas and the whole specimen was sent for frozen section.

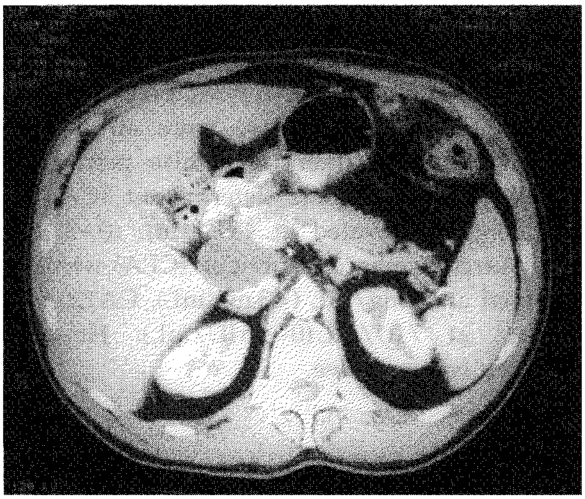

FIGURE 1 CT demonstrating a 51 mm × 35 mm low density, well-defined lesion between the head of the pancreas and the vena cava.

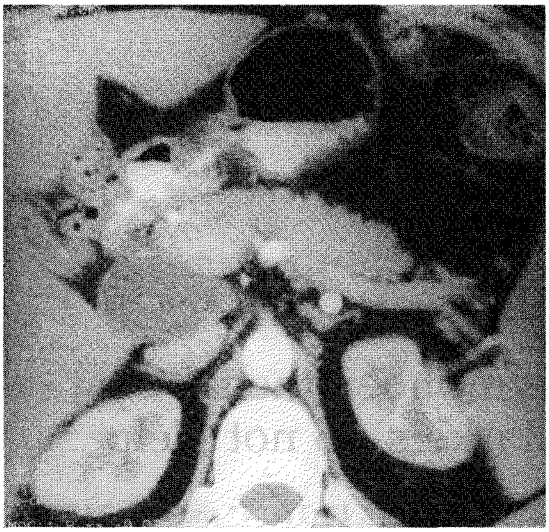

FIGURE 2 Close up view of the lesion on the same CT.

This was reported as a squamous lined non-malignant cyst. A left lateral hepatectomy and cholecystectomy were also performed to deal with the hydatid cyst and gallstones.

Macroscopically, the pancreatic cyst consisted of a slightly lobulated ovoid portion of tissue measuring 65 mm × 50 mm and up to 40 mm in thickness which appeared covered by a thin capsule. On sectioning, the lesion was cystic and contained necrotic soft white material. Microscopically, sections showed a large simple cyst lined by stratified squamous epithelium and containing abundant keratin. The wall of the cyst contained lymphoid tissue with reactive germinal centres and lymphogranulomata. The squamous epithelium was not dysplastic and no appendages were seen. No other epithelial elements were identified. The two other specimens were consistent with a hydatid cyst and chronic cholecystitis.

### DISCUSSION

True cysts of the pancreas are uncommon with pseudocysts accounting for upto 90% of all pancreatic cysts [6]. Lymphoepithelial cysts form a rare subset of true pancreatic cysts. Lüchtrath and Schriefers first reported this lesion in 1985 [16]. Our literature search reveals that only a further 28 cases have been reported [1-5, 7-14, 16-25] (four of these were reported as accessory splenic epidermoids within the pancreas [3, 12, 19, 26]). Truong *et al.*, were the first to give this lesion its current name [22]. With increasing numbers of case reports, characteristics of this rare lesion have become apparent.

The pathogenesis of the lymphoepithelial cyst has not been determined but there are four main current theories [21, 23]. Firstly, it may arise from a branchial cyst fused within the pancreas during embryological development (similar to branchial cysts in the neck). Secondly, the cyst may result from squamous metaplasia of an obstructed pancreatic duct which subsequently protrudes into a peripancreatic lymph node. Thirdly, the cyst may arise from accessory splenic tissue within the pancreas. Lastly, benign epithelial inclusion or ectopic pancreatic tissue in a peripancreatic lymph node may be the origin of this lesion.

The striking feature of this lesion is its histopathology. All the cysts have been spherical, single, well-circumscribed cysts. The cyst contains a white material which is keratin produced by the mature squamous epithelium which lines the cyst. Surrounding this is a layer of lymphoid tissue. The cyst is then separated from the normal pancreatic tissue by a thin capsule of fibrous tissue [23].

The lymphoepithelial cyst predominantly affects males in their middle to late years. At least 8 cases have been found incidentally either on imaging or at autopsy [5, 13, 18, 20, 25]. The rest have presented with non-specific symptoms of abdominal pain, nausea, vomiting, diarrhoea, malaise or weight loss.

Our case has illustrated the difficulty in obtaining a pre-operative diagnosis. There is no blood test specific to this lesion. All the reported cases have shown no consistent pattern in Amylase or tumour markers. Ultrasound, CT and Magnetic Resonance Imaging will all demonstrate a cystic lesion within or near the pancreas. However, none of them are able to differentiate the lymphoepithelial cyst from other cystic lesions of the pancreas. ERCP may help to exclude a pseudocyst, but again does not differentiate between true cystic lesions [9, 21, 24].

### *References*

[1] Bastens, B., Golaire, B., Capelli, Lamy, V., Dryjski, J. and Moisse, R. (1992). Kyste lymphoepithelial du pancreas [Lymphoepithelial cyst of the pancreas]. *Gastroenterol. Clin. Biol.*, **19**, 808-810.

[2] Cappellari, J. O. (1993). Fine needle aspiration cytology of a pancreatic lymphoepithelial cyst. *Diagn. Cytopathol.*, **9**, 77-81.

[3] Davidson, E. D., Campbell, W. G. and Hersh, T. (1980). Epidermoid splenic occurring in an intrapancreatic accessory spleen. *Dig. Dis. Sci.*, **25**, 964-967.

[4] De-Lorenzi, D., Candinas, D., Flury, R., Meyenberger, C. and Largiader, F. (1993). Lymphoepitheliale Zyste des Pankreas [Lymphoepithelial cyst of the pancreas]. *Helv. Chir. Acta*, **60**, 131-135.

[5] DiCorato, M. P. and Schned, A. R. (1992). A rare lymphoepithelial cyst of the pancreas. *Am. J. Clin. Pathol.*, **98**, 188-191.

[6] Fernández-del Castillo, C. and Warshaw, A. L. (1995). Cystic tumors of the pancreas. *Surg. Clin. North Am.*, **75**, 1001-1016.

[7] Fitko, R., Kampmeier, P. A., Batti, F. H., Benjoya, R. A. and Rao, S. M. (1994). Lymphoepithelial cyst of the pancreas with sebaceous differentation. *Int. J. Pancreatol.*, **15**, 145-147.

[8] Goodman, P., Kumar, D. and Balachandran, S. (1994). Lymphoepithelial cyst of the pancreas. *Abdom. Imaging*, **19**, 157-159.

[9] Hausegger, V. K. W., Leitner, G., Wagner, C. and Stangl, F. (1993). Lymphoepitheliale zyste am pankreasschwanz - ein fallbreicht [Lymphoepithelial cyst of the tail of the pancreas - a case report]. *Rofo Fortschr. Geb. Röntgenstr. Neuen Bildgeb. Verfahr*, **159**, 200-202.

[10] Hisaoka, M., Haratake, J., Horie, A., Yasunami, Y. and Kimura, T. (1991). Lymphoepithelial cyst in a 65 year old man. *Hum. Patho.*, **22**, 924-926.

[11] Iacono, C., Cracco, N., Zamboni, G., Bernardello, F., Zicari, M., Marino, F., Montresor, E. and Serio, G. (1996). Lymphoepithelial cyst of the pancreas. Report of two cases and review of the literature. *Int. J. Pancreatol.*, **19**, 71-76.

[12] Jibu, T., Nagai, H., Senba, D., Wada, Y., Kuroda, A., Morioka, Y. and Kikuchi, F. (1987). [A case of epidermoid cyst occurring in an intrapancreatic accessory spleen]. *Nippon Shokakibyo Gakkai Zasshi.*, **84**, 1859-1862.

[13] Kaiserling, E., Seitz, K. H., Rettenmaier, G., Seidel, W., Kahlfuss, R., Walz-Mattmuller, R. and Becker, V. (1991). Lymphoepithelial cyst of the pancreas. Clinical, morphological, and immunohistochemical findings. *Zentralbl. Pathol.*, **137**, 431–438.

[14] Katz, D. S., Scatorchia, G. M., Wojtowycz, A. R. and Botash, R. J. (1995). Lymphoepithelial cyst of the pancreatic head [letter]. *Am. J. Roentgenol.*, **165**, 489.

[15] Lewandrowski, K. B., Southern, J. F., Pins, M. R., Compton, C. C. and Warshaw, A. L. (1993). Cyst fluid analysis in the differential diagnosis of pancreatic cysts. *Ann. Surg.*, **217**, 41–47.

[16] Lüchtrath, H. and Schriefers, K. H. (1985). Pankreaszyste unter Bildeiner sogenannten branchiogen zysterom [A pancreatic cyst with features of a so-called branchiogenic cyst]. *Pathologe.*, **6**, 217–219.

[17] Mitchell, M. L. (1990). Fine needle aspiration biopsy of peripancreatic lymphoepithelial cysts. *Acta Cytol.*, **34**, 462–463.

[18] Mockli, G. C. and Stein, R. M. (1990). Cystic lymphoepithelial lesion of the pancreas. *Arch. Pathol. Lab. Med.*, **114**, 885–887.

[19] Morohoshi, T., Hammato, T., Kunimura, T., Yoshida, E., Kanda, M., Funo, K., Nagayama, T., Maeda, M. and Araki, S. (1991). Epidermoid cyst derived from an accessory spleen in the pancreas. A case report with literature survey. *Acta Pathol. Jpn.*, **41**, 916–921.

[20] Ramsden, K. L. and Newman, J. (1991). Lymphoepithelial cyst of the pancreas. *Histopathology*, **18**, 267–268.

[21] Schinke-Nickl, D. A. and Muller, M. F. (1996). Case report: lymphoepithelial cyst of the pancreas. *Br. J. Radiol.*, **69**, 876–878.

[22] Truong, L. D., Rangdaeng, S. and Jordan, P. H. (1987). Lymphoepithelial cyst of the pancreas. *Am. J. Surg. Pathol.*, **11**, 899–903.

[23] Truong, L. D., Stewart, M. G., Hao, H., Yutani, C. and Jordan, P. H. (1995). A comprehensive characterization of lymphoepithelial cyst associated with the pancreas. *Am. J. Surg.*, **170**, 27–32.

[24] Ueno, S., Muranaka, T., Maekawa, S., Masuda, Y., Ro, T., Saku, M. and Oshimi, Y. (1994). Radiographic features in lymphoepithelial cyst of the pancreas. *Abdom. Imaging*, **19**, 232–234.

[25] Yamamoto, K., Fujimoto, K., Matsushiro, T. and Ota, K. (1990). Lymphoepithelial cyst in the pancreas: a case report. *Gastroenterol Jpn.*, **25**, 758–761.

[26] Yamada, K., Murao, S., Yoshida, H., Nakajima, T., Yoshii, M., Kimura, M. and Yoshioka, H. (1991). [A case of accessory splenic epidermoid cyst]. *Nippon Naika Gakkai Zasshi.*, **70**, 1007–1011.

